# *Ex Vivo* Drug Screening Informed Targeted Therapy for Metastatic Parotid Squamous Cell Carcinoma

**DOI:** 10.3389/fonc.2021.735820

**Published:** 2021-09-16

**Authors:** Noora Nykänen, Rami Mäkelä, Antti Arjonen, Ville Härmä, Laura Lewandowski, Eileen Snowden, Rainer Blaesius, Ismo Jantunen, Teijo Kuopio, Juha Kononen, Juha K. Rantala

**Affiliations:** ^1^Misvik Biology Oy, Turku, Finland; ^2^Department of Oncology and Metabolism, University of Sheffield, Sheffield, United Kingdom; ^3^Genomic Sciences, BD Technologies, Research Triangle Park, Durham, NC, United States; ^4^Central Finland Health Care District, Jyväskylä, Finland; ^5^Department of Biological and Environmental Science, Jyväskylä, Finland; ^6^Docrates Cancer Center, Helsinki, Finland

**Keywords:** *ex vivo* drug screening, precision oncology, HER2, T-DM1, trastuzumab, molecular profiling, parotid squamous cell carcinoma

## Abstract

The purpose of *ex vivo* drug screening in the context of precision oncology is to serve as a functional diagnostic method for therapy efficacy modeling directly on patient-derived tumor cells. Here, we report a case study using integrated multiomics *ex vivo* drug screening approach to assess therapy efficacy in a rare metastatic squamous cell carcinoma of the parotid gland. Tumor cells isolated from lymph node metastasis and distal subcutaneous metastasis were used for imaging-based single-cell resolution drug screening and reverse-phase protein array-based drug screening assays to inform the treatment strategy after standard therapeutic options had been exhausted. The drug targets discovered on the basis of the *ex vivo* measured drug efficacy were validated with histopathology, genomic profiling, and *in vitro* cell biology methods, and targeted treatments with durable clinical responses were achieved. These results demonstrate the use of serial *ex vivo* drug screening to inform adjuvant therapy options prior to and during treatment and highlight HER2 as a potential therapy target also in metastatic squamous cell carcinoma of the salivary glands.

## Introduction

*Ex vivo* drug screening methods in the context of cancer research collectively refer to high-throughput screening (HTS) approaches that utilize vital patient-derived tumor cells as models for assessing drug efficacy. Similarly, as *in vitro* cell-based high-throughput drug screening, *ex vivo* drug screening methods allow assessment of cellular responses to up to thousands of drug perturbations in a single experiment (1–6). The utility of *ex vivo* drug screening has been pioneered in the context of hematological malignancies in which cancer cells can be collected and enriched for HTS directly from blood or bone marrow biopsies (7–10). These studies have demonstrated that the methods can be used to complement pathological cancer diagnostic procedures to track patient-specific drug sensitivity and guide treatment decisions on the most effective treatments or potential alternative therapies currently approved for other cancer indications. The first clinical trial utilizing *ex vivo* chemosensitivity profiling (NCT03096821) to inform treatments of patients with aggressive forms of hematological cancers reported an 88% overall response rate (ORR) in patients matched to treatments on the basis of an *ex vivo* drug screening assay (11). By comparison, in recent clinical trials for genomics matched targeted therapies, the reported ORRs have varied from 11% to 36% (12–15). This suggests that the *ex vivo* drug screening methods could be used to improve the stratification of targeted cancer treatments and complement genomic oncology medicine approaches for personalized care of individual cancer patients. To improve the feasibility and accuracy of *ex vivo* drug screening techniques for solid cancers, especially without the need for invasive surgical tissue sampling, new assay strategies are needed. As an approach for diagnostic therapy efficacy testing in solid cancers, we devised a strategy integrating a phenotypic image-based assay method (1–4) with reverse-phase protein array (RPPA) drug screening to analyze patient-specific therapy efficacy for a rare metastatic parotid squamous cell carcinoma (SCC). Tumor cells isolated from a disease-affected lymph node were analyzed prior to treatment initiation and cells isolated from a distal metastatic lesion were analyzed to adjust treatment strategy after disease recurrence. Altogether, the efficacy of 193 anti-cancer compounds was evaluated to establish a comprehensive chemosensitivity profile. Parotic SCC is a rare, aggressive salivary gland malignancy to which a consensus regarding the use of adjuvant chemotherapy does not exist (16). Moreover, clinical development of novel treatments to this malignancy is limited by the low number of cases, which limits the use of conventional clinical study designs. Therefore, alternative approaches, such as the *ex vivo* screening, are needed to collect evidence on the efficacy of alternative targeted treatment strategies matched to the molecular characteristics of SCC tumors (17).

## Materials and Methods

### Tumor Biopsy Samples

The patient, a 61-year-old male, was identified to the study by an oncologist at the Jyväskylä Medical Centre (Jyväskylä, Finland). The tissue biopsies (surgically resected lymph node, sample A and 3 × 18 gauge needle biopsies, sample B) were collected for the *ex vivo* drug screening with written informed consent from the patient and approval from the local Ethics Committee of the Central Finland Health Care District (KSSHP 3U/2015). All the experiments were undertaken with the understanding and written consent of the patient, and the study methodologies conformed to the standards set by the Declaration of Helsinki.

### Image-Based *Ex Vivo* Drug Screening

The *ex vivo* drug screens were performed as previously described (3). Briefly, the therapeutic compound collection used in the drug screening consisted of 125 (sample A) and 193 (sample B) anti-cancer agents, purchased from commercial chemical vendors (Selleck Biochemical, Santa Cruz Biotechnology). To allow maximally broad characterization of different drug classes with the limited number of cells available from the tumor biopsies, each compound was tested in five different concentrations with twofold (sample A) and threefold (sample B) dilutions starting from 5 μM as the highest concentration. The single-cell milieu of freshly isolated tumor-derived cells (45 µl per well; 1,000 cells per well) was transferred to each well using a peristaltic MultiDrop Combi dispenser (ThermoScientific). The 384-well plates were incubated for 96 h in standard cell culture conditions, 37°C and 5% CO_2_. Analysis of cell viability with cellular lineage separation was performed through high-content imaging. The cell cultures were fixed with 4% paraformaldehyde with 0.03% Triton-X100 and incubated overnight at +4°C with antibodies against epithelial cytokeratin-19 (KRT19, Abcam, Clone EP1580Y), stromal cell marker vimentin (VIM, Santa Cruz Biotechnology, Clone V9), and HER2 (DAKO, A0485). Secondary antibody staining was performed at room temperature for 1 h with AlexaFluor secondary antibodies against the primary host species (1:500, LifeTech) in 1% BSA. DAPI (4′,6- Diamidino-2-phenylindole nuclear counterstain, LifeTech) (1 μg/ml) was added to secondary staining buffers for DNA counterstaining. Cells were imaged using Olympus scan^R platform at 20× magnification. Nine frames were acquired from each 384-well to cover the whole well area. Images were analyzed with Olympus scan^R image analysis suite including integrated DNA staining-based primary object segmentation using a watershed algorithm. Primary objects (nuclei) were expanded a fixed 20-pixel distance, and mean fluorescence signal intensity for KRT19 and VIM was quantified from this expanded cellular region. Single-cell positivity for KRT19, VIM, and HER2 was determined by gating in the scan^R image analysis suite, using secondary antibody only stained cells for each marker as controls. Population separated cell count data were normalized using the GR method (18) (see Equation 1 in *Statistical Analysis*) to DMSO-only wells (negative control), 5 µM staurosporin-containing wells (positive control), and 2 µM aphidicolin-containing wells (cell growth control). Dose–response curves and growth rate normalized IC_50_ estimates were generated in GraphPad Prism software (V8, GraphPad Software). The *ex vivo* drug screening data (**Supplementary Figures S1****–****S3**) are deposited to Mendeley data (DOI: 10.17632/9t7gn926ry.1).

### Targeted Genomic Sequencing

The genomic profiling was performed at the Jyväskylä Medical Centre molecular pathology core (Jyväskylä, Finland). Briefly, genomic DNA was extracted from the coarse needle tissue biopsy with QIAamp DNA Mini Kit (Qiagen) according to the protocol provided by the kit manufacturer. Qiaseq Human Comprehensive Cancer Panel (Qiagen, DHS-3501Z) including 275 cancer-related genes was used to prepare NGS amplicon gene library according to the protocol provided by the kit manufacturer. Unique molecular identifiers (UMIs) were used to tag individual DNA strands. Sequencing was performed with Illumina NextSeq500 instrument (Illumina) according to standard protocol. Data were de-multiplexed and fastq files were created with bcl2fastq software (Illumina). The data were processed in CLC Biomedical Genomics Workbench (Qiagen) with workflow provided by Qiagen and using Hg19 human reference genome to call the gene variants. Gene annotations were performed according to the vcf files in OmnomicsNGS software (Euformatics, Espoo, Finland). The sequencing data files are deposited to NCBI Sequence Read Archive (SRA) under accession no. PRJNA760256.

### Immunohistochemistry

Immunohistochemical stainings were performed by following standard procedures. Shortly, 2-µm FFPE sections were stained with Bond-III automated IHC stainer (Leica Biosystems) and Bond Polymer Refine Detection kit (DS9800, Leica Biosystems). For BLC-2, antigen retrieval was performed with Bond Epitope Retrieval Solution 2 (AR9640, Leica Biosystems) at 100°C for 20 min. The used antibody dilutions were 1:100 for KRT19 (clone A53-B/A2.26, ThermoScientific), VIM (clone V9, Novocastra/Leica), and HER2 (clone SP3, ThermoScientific). Thirty minutes incubation time was used for all antibodies. All stainings were interpreted by a pathologist.

### HER2 Dual ISH Assay

HER2 amplification assay to formalin-fixed paraffin-embedded sections was done with fully automated Ventana BenchMark Xt slide stainer (Ventana Medical Systems). Shortly, dinitrophenyl (DNP)-labeled HER2 probe and digoxigenin-labeled chromosome 17 (Chr17) probes (INFORM HER2 Dual ISH DNA Probe Cocktail, Roche) were co-hybridized to their targets. HER2-DNP probe was visualized with UltraView SISH DNP detection kit (Roche) using HRP-driven silver metallographic detection. For Chr17-DIG probe, UltraView Red ISH DIG detection kit (Roche) was used to produce red signal in alkaline phosphatase-driven reaction. The copy number of HER2 was counted from minimum 20 representative nuclei. HER2 copy number ≥6 was determined as positive for amplification. If HER2 copy number was uncertain (between 4 and 6), the number of Chr17 centromeres was also counted. HER2/Chr17 ratios <2 were interpreted as non-amplified and ratios ≥2 were interpreted as amplified.

### RPPA, Quantitation, and Analysis

For the RPPA, screening cells (1,000 per well) were treated in 384-microplate wells with 19 drugs in five concentrations with twofold dilutions starting from 5 µM as the highest concentration. Lysates were collected at the 48-h time point after drug treatment. To generate reverse-phase arrays, lysates were printed on nitrocellulose-coated glass slides (Grace Biolabs #305177) on a Genetix QArray Mini Arrayer (Molecular Devices). Primary antibodies used for RPPA experiments contained the following: p-AKT (Ser473) (Cell Signaling Technology Cat# 4060), AKT (Cell Signaling Technology Cat# 4691), p-ERK1/2 (Thr202/Tyr204) (Cell Signaling Technology Cat# 4370), p-S6 Ribosomal protein (Ser235/236) (Cell Signaling Technology Cat# 2211), Ki-67 (Abcam Cat# ab15580), HER2 (DAKO Cat# A0485), p-HER2 (Y1248) (R&D Systems Cat# AF1768), and p-cMET (Tyr1234/1235) (Cell Signaling Technology Cat# 3129). Secondary detection was performed with a goat anti-mouse IgG conjugated to DyLight 680 (Thermo Pierce Cat# 35518) and goat anti-rabbit IgG conjugated to DyLight 800 (Thermo Pierce Cat# 35571) antibodies. For total protein measurement, the arrays were stained with a Sypro Ruby Blot solution (Invitrogen) and an antibody for actin (Cell Signaling Technology Cat# 4970). The slides were scanned with a Tecan LSReloaded (Tecan) microarray scanner to detect the Sypro signals and Odyssey Licor IR‐scanner (LI‐COR Biosciences) to detect the antibody signals. Array‐Pro Analyzer microarray analysis software (V6.3 Media Cybernetics) was used for analyzing the data. Antibody signals were normalized to the Sypro total protein, log transformed, and *z*‐score standardized. *z*‐scores < −2.0 or >2.0 (−/+ 2×standard deviation from the whole screen) were considered as significantly downregulated or upregulated, respectively. Box plots, heatmaps, and Pearson correlation analyses were created on GraphPad Prism software.

### Flow Cytometry

MISB10 cells cultured in T75 flasks were treated with Accutase (BD Biosciences) and washed twice with PBS with 1% BSA. Suspensions were counted and measured for cell viability using the Vi-Cell XR cell counting and viability analyzer (Beckman Coulter). Cells were diluted in PBS to 1 × 10^6^ cells/ml and stained for 30 min at RT with membrane viability dye (LIVE/DEAD Fixable Near-IR, Invitrogen). Cells were washed and distributed to a 96-well plate containing staining antibodies and Hoechst 33342 (Invitrogen). For characterization panels, immunophenotyping antibodies to the following targets were used: HER-2/neu, CD24, CD29, CD44, CD45, CD49f, CD90, CD166, CD326 (BD Biosciences), and CD133 (Miltenyi Biotec, Auburn, CA). Cells were incubated for 30 min at RT in the dark and then rinsed twice in PBS before acquisition with a BD LSRII flow cytometer. Analysis of results was performed using FACSDiva v6.1.3 and FlowJo analysis software (FlowJo).

### MISB10 Cell Line

Approval to use the cells for culture and *in vitro* research was obtained from the patient and the local ethical committee prior to the study. The tumor tissue-derived cells were grown for 4 months in RPMI-1640 culture media supplemented with penicillin/streptomycin (100 units/100 mg), l-glutamine (2 mmol/L), fetal bovine serum (5%, Biowest), and 1× ITS-G (Insulin-Transferrin-Selenium, Gibco). The resulting cell line, designated MISB10, grew continuously when the culture medium was changed twice per week. Confluent cultures were dissociated for 2 min at +37°C using TrypLE Select enzyme (Gibco), and split into new cultures with ratios of 1:2 or 1:3. Cryopreservation was done in CellVation (MP Biomedicals) cryopreservation media. The MISB10 cell line will be available for non-profit research purposes through the corresponding author on reasonable request and in accordance to a specified MTA.

### Statistical Analysis

The *ex vivo* drug screening data were analyzed using the normalized growth rate inhibition (GR) approach, which yields per-division metrics for drug potency and efficacy. The GR values were calculated from the raw image cytometry cell count data with no plate normalization or other data pre-processing and used for comparison of drug potency between the epithelial and stromal sub-cell populations having a differential proliferation rate during the screening. GR values were calculated using:

– *x*(*c*), the cell count value of a cell population per well following drug treatment at concentration *c*.
– *x_ctrl_*, the average cell count value of a cell population in DMSO-treated control wells from the same plate; *x_ctrl_* = mean({*x_i_*∈*x* | abs(log10(*x_i_*)–log10(mean(*x*)))o1.5)}), where x are all DMSO-treated control values.
– *x_0_*, the average cell count value of a cell population in 2 µM aphidicolin-treated control wells from the same plate; *x_0_ *= mean({*x_i_*∈*x* | abs(log10(*x_i_*)–log10(mean(*x*))) o1}), where *x* is the vector of treated values.

**Supplementary Data 1****,****2** comprise the GR values for each treated condition of the KRT19^+^, VIM^+^, and HER2^+^ cell populations calculated as follows:


(1) $$GR(c)=\frac{\log_{2}(X(c)/X_{0}}{2^{\log_{2}\left(\frac{X_{ctrl}}{X_{0}}\right)}-1}$$

Welch’s *t*-test, Student’s *t*-test, and Pearson correlation analyses as indicated in the figure legends were applied using GraphPad Prism V8 software according to assumptions on data normality.

## Results

### *Ex Vivo* Drug Screen on Lymph Node Metastasis-Derived Tumor Cells

**Figure 1:**
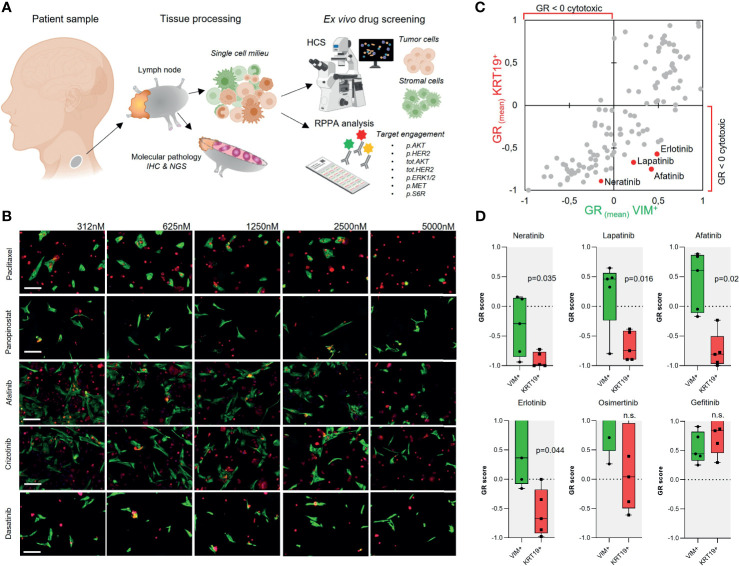
*Ex vivo* drug screening in metastatic parotid duct carcinoma. **(A)** Schematic presentation of the study strategy. **(B)** Gallery view of representative 20× immunofluorescence microscopy images of the sample cells treated for 96 h with the indicated drugs in five different concentrations (nM). KRT19 staining shown in red and VIM staining in green. Bars: 50 µm. **(C)** Scatter plot showing correlation of the mean GR score of 125 drugs in five doses for the KRT19^+^ and VIM^+^ cells. **(D)** EGFR-TKis were identified as drugs with a selective cytotoxic effect on the KRT19^+^ cells. Box plots showing the GR scores for the five doses of all EGFR-TKi included in the screen and compared using paired Student’s *t*-test.

Our patient, a 61-year-old male, originally presented with facial nerve paresis and expansive mass in neck 3 years earlier to the study. Primary diagnostic studies revealed a mass in his left parotid gland. Following a lung CT, a radical parotidectomy and dissection of neck lymph nodes to regions I–V was performed. Histopathological diagnosis was poorly differentiated squamous cell cancer of the parotid gland and post-operative PET-CT scan revealed multiple lung metastases and residual activity in the tumor bed area. Treatment of the disease was initiated with cisplatin–5-fluorouracil chemotherapy regimen to reduce tumor burden. After two cycles of chemotherapy, concurrent chemoradiation therapy with weekly cisplatin was initiated and completed to 50 Gy dose to the residual tumor operation bed and left neck lymph node areas. Local control of the disease was achieved, but lung metastases remained, and residual lung lesions were considered inoperable. Following a 3-month treatment holiday, chemotherapy was changed to nab-paclitaxel (Abraxane). CT scan for response evaluation showed stable disease after 4 months. Response evaluation at 12 months showed progressive disease. At this time, as per discussion with the patient and with approval from the local Ethics Committee of the Central Finland Health Care District, a lymph node sample was retrieved from the left axilla area and processed for the *ex vivo* screening under written informed consent by the patient. The node was cut in half and one-half was processed for routine histopathology. The inner mass of the other half was drained from excess immune cells, cut into small fragments, and disaggregated enzymatically to achieve a single cell milieu of cells (1) (**Figure 1A**). The resulting cell suspension was diluted into cell culture medium and dispensed immediately onto drug containing 384-microwell plates (**Supplementary Figure S1**). Following 96-h exposure of the cells to 125 drugs in five concentrations, high-content imaging cytometry with immunofluorescent antibody staining of the cells with cytokeratin-19 (KRT19) as epithelial (19) and vimentin (VIM) as stromal (1, 3) cell marker was used to quantify the cell-type-specific drug efficacy (**Supplementary Figure S1A**). Dose responses were compared to the negative and positive assay controls to normalize the dose responses of the two cell populations separately to the measured growth rate (3, 18) of each population as described in Equation 1; 77.1% of all analyzed cells were KRT19^+^ and 19.5% were VIM^+^. The calculated cell doubling rate of the KRT19^+^ cells was ~220 h and ~135 h for the VIM^+^ cells, corresponding to 0.44 and 0.77 cell divisions over the course of the 96-h assay, respectively (18) (**Supplementary Figure S1B**). To identify the most potent tumor cell-targeted cytotoxic drugs, we compared the GR-corrected IC_50_ estimates of the drugs between the KRT19^+^ and VIM^+^ cells (**Figure 1C** and **Supplementary Figure S2A**). Topoisomerase inhibitors, HDAC inhibitors, alkaloid and taxane tubulin poisons, CDK inhibitor dinaciclib, and TKi dasatinib were collectively the most cytotoxic drugs on both cell types with an average IC_50_ below 1 µM (**Figure 1B** and **Supplementary Figure S2B**). Twenty-two drugs had a selective cytotoxic effect only on the KRT19^+^ cells (**Figure 1C**). The EGFR-TKis afatinib, erlotinib, and neratinib, AKT-HER2 inhibitor lapatinib, and c-METi crizotinib were the most potent drugs with a selective cytotoxic effect only on the KRT19^+^ cells (**Figures 1B–D**).

### *Ex Vivo* Informed Treatment

**Figure 2:**
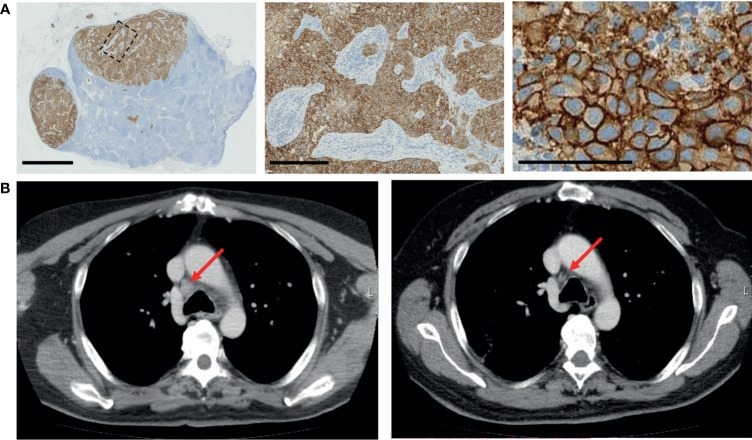
Validation of HER2 status and targeted therapy. **(A)** Immunohistochemistry validation of HER2 staining in the affected lymph node. Scale bars: 2.5 mm, 250 µm, and 100 µm (left to right). **(B)** Radiologic response of patient. Left, prior to treatment, and right, after four cycles of treatment with T-DM1.

Based on the *ex vivo* drug screening results, the patient’s tumor cells were selectively most sensitive to EGFR/HER2 inhibitors as a class of drugs. Amplifications, high expression levels, and genomic aberrations of HER2 have been reported frequent in a subset of salivary duct carcinomas (17, 20) and several case studies using EGFR/HER2-TKi therapies including cetuximab, erlotinib, gefitinib, T-DM1, and trastuzumab on individual salivary gland cancer patients have been reported. However, at the time of diagnosis of the metastatic disease, checking HER2 status was not considered standard clinical practice in parotid carcinoma in Finland. To assess whether our patient’s tumor cells’ sensitivity to the EGFR/HER2 inhibitors was due to possible amplification of HER2, additional histopathological analysis including HER2 immunohistochemistry and dual ISH HER2 amplification assay was performed on the lymph node tissue from which the sample cells to the *ex vivo* assay were isolated. Immunohistochemistry results indicated complete, strong, membranous staining, compatible with 3+ HER2 (ASCO/CAP HER2 expression criteria) (**Figure 2A**) accompanied with a copy number gain confirmed with CISH analysis. Based on the *ex vivo* drug screening result, the immunohistochemistry confirmation and an earlier case report describing sustained clinical response of two HER2^+^ metastatic salivary gland cancer to ado-trastuzumab emtansine (T-DM1) (21, 22), the patient was considered for treatment with T-DM1. In Finland, HER2-directed therapies are still not reimbursed or generally available for salivary gland cancers. However, after clinical evaluation, treatment with T-DM1 was initiated with off-label use. After 4 months and four cycles of T-DM1, a partial response was confirmed with radiographic examination (**Figure 2B**). The patient was asymptomatic and tolerated the treatment well. After 6 months, the lung and lymph node lesions continued to shrink. Systemic treatment with T-DM1 was continued, and at 10 months of therapy, lung lesions remained stable, but a new 4-cm tumor lesion had appeared between the sixth and seventh rib and the lumbar spine (L1). A new biopsy sample for repeated *ex vivo* analysis was then retrieved from the lesion between the ribs (**Supplementary Figure S3**).

### Repeat Analysis of Drug Efficacy in Recurrent Disease

**Figure 3:**
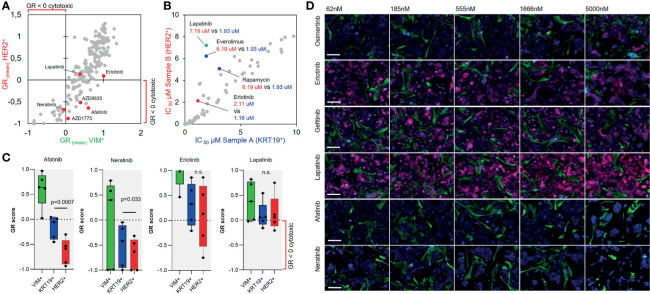
Repeat *ex vivo* drug screen in recurrent disease. **(A)** Scatter plot showing correlation of the mean GR score of 193 drugs in five doses for the HER2^+^ and VIM^+^ cells. **(B)** Scatter plot showing correlation of the IC_50_ estimate of the analyzed drugs between the HER2^+^ sample B cells and KRT19^+^ sample A cells. **(C)** Box plots showing the GR scores for the five doses of afatinib, neratinib, erlotinib, and lapatinib in sample A and B KRT19^+^ and HER^+^ cells. Afatinib and neratinib displayed a statistically significant differential effect on the KRT19^+^ and HER^+^ cells from sample B as compared using paired Student’s *t*-test. **(D)** Gallery view of representative 20× immunofluorescence microscopy images of the sample cells treated for 96 h with the different EGFR-TKis in five different concentrations (nM). KRT19 staining shown in blue, VIM staining in green and HER2 staining in red. Bars: 50 µm.

To evaluate drug sensitivity of the new metastatic lesion and the recurrent disease, a repeat *ex vivo* drug screening was undertaken with cells isolated from the new lesion. A parallel tissue biopsy from the same lesion was subjected to targeted DNA sequencing. The image-based assay strategy as used with sample A was used again with staining of HER2 as a third marker for detection of HER2^+^ cells (**Figure 3D**). Efficacy of 193 drugs in five doses was compared between the VIM^+^, KRT19^+^, and HER2^+^ cells (**Supplementary Figures S1** and **S4A**). One hundred percent of the KRT19^+^ cells were also HER2^+^ (without treatments), and similarly as in sample A analysis, the EGFR-TKis afatinib and neratinib were among the most potent drugs with a selective cytotoxic effect on the KRT19^+^ cells (**Figures 3A, B**). However, the cytotoxic effect of EGFR/HER2 targeted drugs erlotinib and lapatinib, as well as mTOR inhibitors everolimus and rapamycin varied significantly between samples A and B (**Figure 3C** and **Supplementary Figures S4B, C**). MTOR inhibitors AZD2014 and AZD8055, and PI3K inhibitors Buparlisib and AZD8835, on the other hand, showed higher efficacy in sample B (**Supplementary Figures S4B, C**).

**Figure 4:**
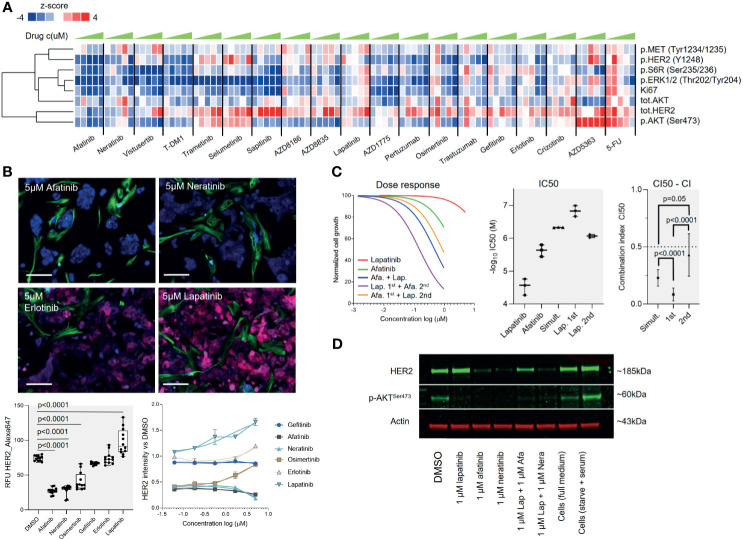
A pathway perspective on targeted therapy responses. **(A)** Heat map visualization with one-dimensional (vertical) unsupervised clustering of the *z*-scores of the eight RPPA markers across 19 drugs in five concentrations (left to right, low to high, respectively). **(B)** Top: Representative microscope images of HER2 staining on sample B cells in the *ex vivo* screen following 96-h exposure to the indicated drugs at 5 µM concentration. KRT19 staining shown in blue, VIM staining in green, and HER2 staining in red. Bars: 50 µm. Bottom left: Box plot showing the mean and standard deviation of fluorescence intensity of HER2 staining (RFU) of the KRT19^+^ cells in response to treatment with the different EGFR-TKis. Two biological replicate experiments were combined and compared using unpaired Welch’s *t*-test. Bottom right: Comparison of the HER2 staining intensity to DMSO-treated cells. Signal shown as fold change. **(C)** Combination of lapatinib and afatanib shows a synergistic growth inhibitory effect. With all three different drug schedules tested, the combination resulted in significantly increased efficacy over the single agents as indicated by the lower IC_50_ (middle panel). On the basis of the CI_50_ combination index, lapatinib pre-treatment preceding afatinib treatment had the highest synergy (right panel). Comparison was made using unpaired Student’s *t*-test. **(D)** The patient-derived tumor cells were treated with DMSO (−), 1000 nmol/L afatinib, lapatinib, neratinib, or combination of 1000 nmol/L afatinib+lapatinib, or neratinib+lapatinib for 48 h, and phosphorylation (p) AKT and total HER2 protein levels of indicated markers were assessed using Western blot. Actin was assessed as a loading control.

To analyze the pathway inhibition efficacy of the most potent identified targeted inhibitors on downstream targets of the HER2/PI3K/AKT/mTOR and EGFR/RAS/MEK pathways, we performed a RPPA screen with the patient-derived cells. In the RPPA experiment, the cells were exposed for 48 h to 16 drugs selected from the *ex vivo* screen including all the EGFR-TKis and trastuzumab, T-DM1 (Kadcyla), and sapitinib (pan-EGFRi) as additional EGFR-TKis. Drugs were tested in five doses with twofold dilutions and eight markers; phospo-S6RP^S235/236^, HER2, phospho-HER2^Y1248^, AKT, phospho-AKT^S473^, phospho-ERK1/2^T202/Y204^, phospho-MET^Y1234/1235^, and Ki-67 were used as the assay readout (**Figure 4A**). Analysis of the RPPA data (**Supplementary Figure S5A**) indicated strongest correlation between the proliferation of the cells (*z*-score Ki-67) and p.HER2 (Pearson correlation, *r* = .43), p.S6R (*r* = .48), and p.ERK1/2 (*r* = .68) (**Figure 4A**). EGFR-TKis including afatinib, sapitinib, and T-DM1 effectively blocked all of these markers, while the dual MTORC1/2 inhibitor AZD2014 (vistusertib) had the strongest dose-dependent effect on MTORC1/2 downstream effectors AKT and S6RP (**Supplementary Figure S5B**).

Supported by the RPPA results and the image-based screen results indicating that the HER2 expression was amplified in a dose-dependent manner by lapatinib and downregulated by the EGFR-TKis (**Figure 4B** and **Supplementary Figure S5A**), we rationalized that combination of lapatinib could potentiate the overall therapeutic efficacy of the EGFR-TKis as reported in several human cancers (23–25). We explored the effect of combining the highest-ranking EGFR-TKi afatinib with lapatinib to evaluate synergy between the two drugs. Three different drug combination schemes were compared with a fixed 1:5 molar ratio of the drugs (afatinib IC_50_ 1.64 µM, lapatinib IC_50_ 7.19): (1) 72-h simultaneous treatment, (2) 24-h lapatinib pre-treatment followed with 48 h addition of afatinib, and (3) 24-h afatinib pre-treatment followed with 48 h addition of lapatinib (**Figure 4C**). The combinations resulted in significant synergistic effects across all three treatment schedules with a mean CI50 combination index (26) of 0.47 with the simultaneous treatment, 0.15 with lapatinib pre-treatment, and 0.86 with afatinib pre-treatment (**Figure 4C**). The impact of the drug combination on HER2 protein level expression and phosphorylation of AKT was also confirmed with a Western blot analysis from the cells (**Figure 4D**). However, the synergistic effect was significantly more potent with lapatinib being administered first in comparison to simultaneous or reversed administration (*p* <.0001 both) (**Figure 4C**), suggesting that the order of administration of the two drugs could affect the overall combination efficacy.

### Treatment of the Recurrent Disease

**Figure 5:**
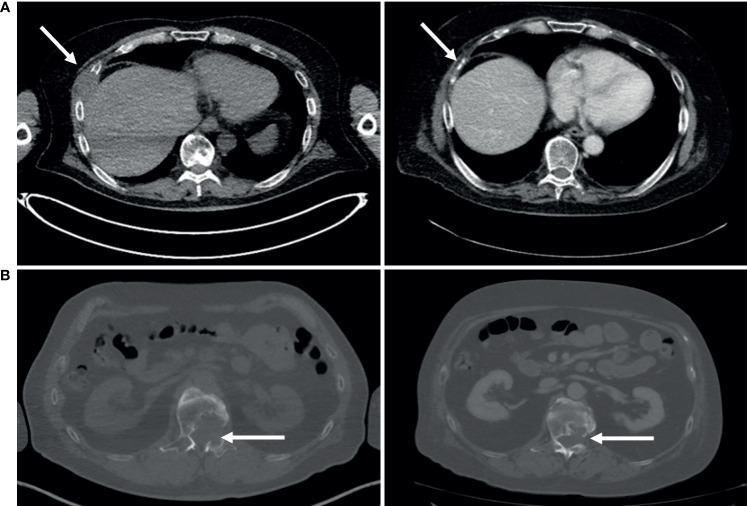
Clinical response to dual inhibitor lapatinib (AKTi)–trastuzumab (HER2i) therapy. **(A)** Following 2 months of treatment, the soft tissue component of the metastasis lesion between the sixth and seventh rib was completely resolved**. (B)** The lytic lumbar spine (L1) lesion was stabilized following 2 months of treatment.

The targeted DNA sequencing performed from sample B identified a p53 p.R273C (allele frequency 69%) mutation, a CHEK2 p.I157T (freq. 40%) mutation, and a HER2 p.V747L mutation (freq. 93%), while HER2 CISH assay confirmed a HER2 copy number gain. Following relapse of the disease after treatment with T-DM1, the patient’s treatment was continued with capecitabine as a single agent. After 2 months, the patient suffered a seizure and multiple brain metastases were detected in MRI. These lesions were not suitable for stereotactic radiotherapy and the patient received palliative whole-brain radiotherapy. The patient was then considered for a second cycle of experimental therapy based on the *ex vivo* drug screening and molecular pathology results. As the *ex vivo* experiments suggested that the patient’s tumor cells continued to respond to EGRF-TKis and that lapatinib potentiated these effects, trastuzumab–lapatinib regimen was initiated (27, 28) with five daily doses of lapatinib as a pre-treatment. Control CT scan performed after two cycles of trastuzumab and daily dosing of lapatinib showed that the lung metastases had remained stable. The soft tissue tumor component of the metastasis between the sixth and seventh rib had been completely resolved with only a sclerotic bony lesion remaining while the lytic, metabolically active metastatic lesion on the lumbar spine (L1) had been stabilized (**Figure 5**). The trastuzumab–lapatinib regimen was continued as therapy for 10 months until clear progressive disease was observed, and the patient started to receive the best palliative care. The patient succumbed to the disease 26 months after the initial *ex vivo* sample was obtained and 60 months from the primary diagnosis.

### A New HER2^+^ Parotid Carcinoma Model Cell Line

**Figure 6:**
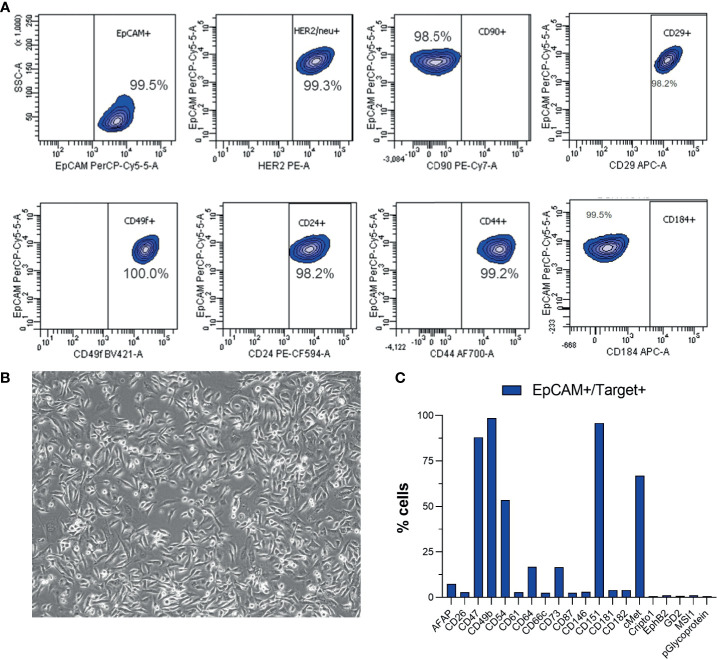
Flow cytometry immunophenotyping of the MISB10B cells. **(A)** Analysis of expression of epithelial lineage markers and HER2 in the EpCAM^+^ cells. **(B)** Representative bright-field microscopy image (10×) of the morphology of the MISB10B cells in near full confluent state. **(C)** Analysis of expression of additional immunophenotypic markers in the EpCAM^+^ cells.

Many of the defined molecular subtypes of salivary gland malignancies represent a HER2^+^ molecular background (20), yet no HER2^+^ model cell lines have been described, and only two salivary gland cancer-derived model cell lines are altogether included in the CCLE collection of 1,739 human cancer cell lines (29). The patient-derived cells left over from the performed *ex vivo* analyses of sample B were kept in culture in standard cell culture conditions after the patient was started treatment with the trastuzumab–lapatinib regimen. After 4 months in culture, the cells started to show stable *in vitro* growth and a uniform morphology with no residual stromal cell contamination growing in the culture. The cells grew continuously as an adherent monolayer (**Figure 6B**) and reached confluence in 4 to 6 days with 1:3 ratio passaging in 10-cm culture dishes. After the cells had undergone ~20 passages, a comprehensive flow cytometry immunoprofiling was performed to establish an immunophenotypic profile of the cells and to assess clonal heterogeneity of the culture. Over 99% of the cells were found to be EpCAM^+^ with >98% being HER2^+^, CD24^+^, CD44^+^, CD29^+^, CD49f^+^, CD90^-^, and CD184^-^ (29) (**Figure 6A**). In addition to these epithelial cell lineage markers, the EpCAM^+^ cells were also found to express CD47, a potential immune evasion marker (30), CD54 (31), CD64, CD73 (32), CD151, and c-MET (**Figure 6C**), which could explain the cells’ responsiveness to the c-METi crizotinib (**Figure 1B** and **Supplementary Figure S2B**). Currently, subcultures of the cells devoted a cell line named MISB10, which have undergone an excess of 80 passages; they show continuous growth and can recover from repeated cryopreservation cycles; they show responsiveness to EGFR-TKi targeted therapies and form multicellular organotypic spheroids while grown in human tumor microenvironment mimicking 3D culture conditions (33) (**Supplementary Figure S6**).

## Discussion

Parotid squamous cell carcinoma is a rare, aggressive salivary gland malignancy, which often presents at an advanced stage with nodal metastases. While most salivary gland tumors in general are benign in nature, this tumor is highly aggressive and approximately 65% of patients die from progressive disease within 48 months (34). There are no standard treatment options for recurrent and metastatic disease, and the role of adjuvant chemotherapy on overall survival is not known due to the very low incidence rate. Surgical resection followed by radiation therapy thus remains the main management strategy for parotid squamous cell carcinomas with limited other therapeutic options for metastatic disease. While up to 44% of parotid duct carcinomas have been reported to have *HER2* amplifications or high protein or mRNA level expression of HER2 (20, 35), the number of HER2-positive parotid SCC cases is lower (36). This suggests that while less frequent in parotid SCC than parotic duct carcinomas, HER2 aberrations represent a significant target, biomarker, and opportunity for targeted treatment also in a subset of patients with parotid SCC tumors (17, 20, 33, 37). To improve outcomes and efficacy of treatment of primary and/or metastatic parotid SCC, systematic studies involving larger series of HER2^+^ salivary gland cancers or stratification of patients to HER2-targeted therapies by alternative strategies are needed to determine the contribution of HER2 targeting on tumor response outcomes in parotid SCC and other salivary gland cancers (17).

In this study, we identified the sensitivity and HER2 positivity of the patient’s cancer on the basis of drug sensitivity of the vital tumor cells to EGFR-TKi in an *ex vivo* drug screen. At time of the study, analysis of HER2 status was not considered standard clinical practice in parotid carcinoma in Finland. This shows that *ex vivo* drug screening can be used as a platform to complement molecular pathology information and quickly identify actionable drug sensitivities that can be matched with the molecular characteristics of the patient’s tumor and thus motivate personalized medicine. By identifying the response of the patient-derived tumor cells to the EGFR-TKis, our results helped to guide our patient’s treatment to include treatment options that are currently still not generally available for these patients, but which resulted in sustained clinical benefit. Molecular profiling of the patient’s tumor also identified a novel *HER2* mutation affecting the protein tyrosine kinase domain and a *CHEK2* mutation previously identified as a cancer susceptibility gene in the Finnish population (38, 39). As *HER2* mutations have been shown to reduce the efficacy of therapies commonly used to treat HER2-positive breast cancer, particularly in metastatic and previously HER2 inhibitor-treated patients (40), our data, even though limited by being an analysis only of one patient, provide novel insights into general and EGFR-TKi-specific drug efficacy in cancers bearing these aberrations. For further analyses of these aspects, the MISB10 cell established from the patient’s tumor represents a unique new *in vitro* research resource as the first and only described *HER2*-amplified, *HER2* mutant salivary gland cancer cell line in the world (41).

In summary, we present here the first large-scale *ex vivo* drug screening in a metastatic parotid squamous cell carcinoma together with use of HER2-targeted therapies adding one more case example to the existing medical literature supporting the use of HER2-directed therapies for a subset of salivary gland tumors. Since parotic SCC is a rare tumor type, it is difficult to conduct prospective clinical studies to compare which of the existing HER2 directed agents is the most effective, but this case example suggests that antibody–chemotherapy conjugates seem to have promising activity. Future studies should also investigate trastuzumab–deruxtecan for this patient population as, currently, there are no adjuvant treatment options that affect the overall survival of metastatic parotid SCC patients (42). New treatment modalities are therefore urgently needed to improve the outcome of this aggressive disease.

## Data Availability Statement

The data presented in the study are deposited in the NCBI Sequencing Read Archive, accession number PRJNA760256 and Mendeley Data repository, accession number DOI: 10.17632/9t7gn926ry.1.

## Ethics Statement

The studies involving human participants were reviewed and approved by Keski-Suomen sairaanhoitopiiri, Tutkimuseettinen toimikunta, Jyväskylä, Finland. The patients/participants provided their written informed consent to participate in this study.

## Author Contributions

Conception and design: RM, AA, JK, and JR. Clinical materials: IJ, TK, and JK. Development of methodology: RM, AA, and JR. Acquisition of data: NN, RM, AA, VH, and LL. Analysis and interpretation of data: LL, RM, IJ, TK, JK, and JR. Writing, review, and/or revision of the manuscript: NN, RM, TK, JK, and JR. Administrative, technical, or material support: TK, JK, and JR. Study supervision: JR. All authors contributed to the article and approved the submitted version.

## Funding

This work has been supported in part by AZ-SLL-KI open innovation grant #18122013. The funding body had no input in the design of the study, collection, analysis, or interpretation of the data.

## Conflict of Interest

Authors NN and JR are employed by Misvik Biology Oy. Authors RM, AA, VH, and LL were employed by Misvik Biology Oy. JR is the founder and owner of Misvik Biology Oy. Authors ES and RB were employed by BD Technologies.

The remaining authors declare that the research was conducted in the absence of any commercial or financial relationships that could be construed as a potential conflict of interest.

## Publisher’s Note

All claims expressed in this article are solely those of the authors and do not necessarily represent those of their affiliated organizations, or those of the publisher, the editors and the reviewers. Any product that may be evaluated in this article, or claim that may be made by its manufacturer, is not guaranteed or endorsed by the publisher.
