# Supplementary figures and images for: Ex Vivo Drug Screening Informed Targeted Therapy for Metastatic Parotid Squamous Cell Carcinoma
Source: Front Oncol. 2021 Sep 16;11:735820. doi: 10.3389/fonc.2021.735820 (PMC8481915; doi:10.3389/fonc.2021.735820)

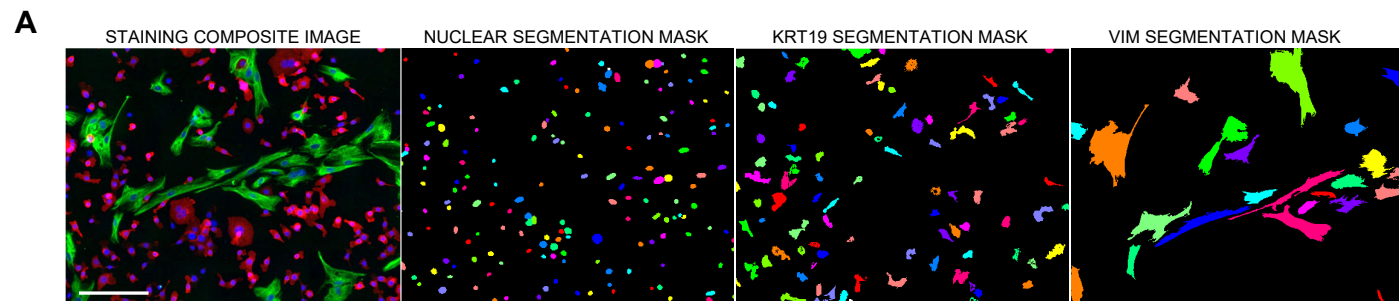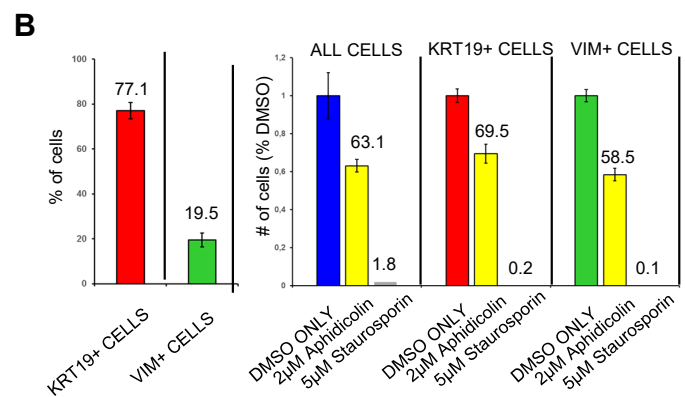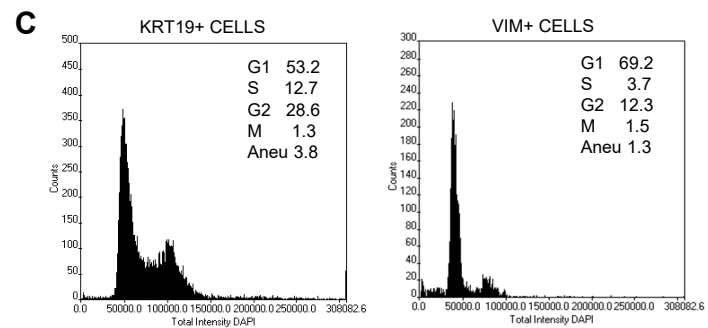

Supplement: Supplementary file 1 [file DataSheet_1.pdf]

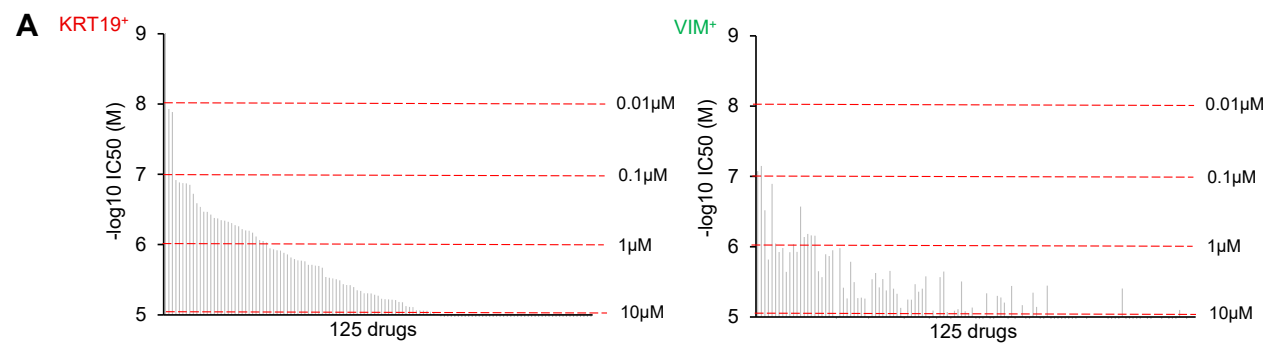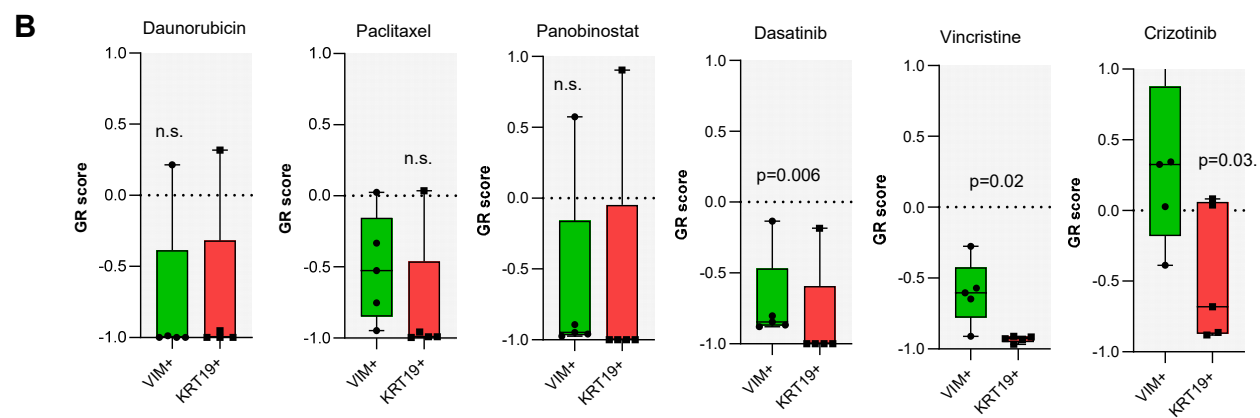

Supplement: Supplementary file 2 [file DataSheet_2.pdf]

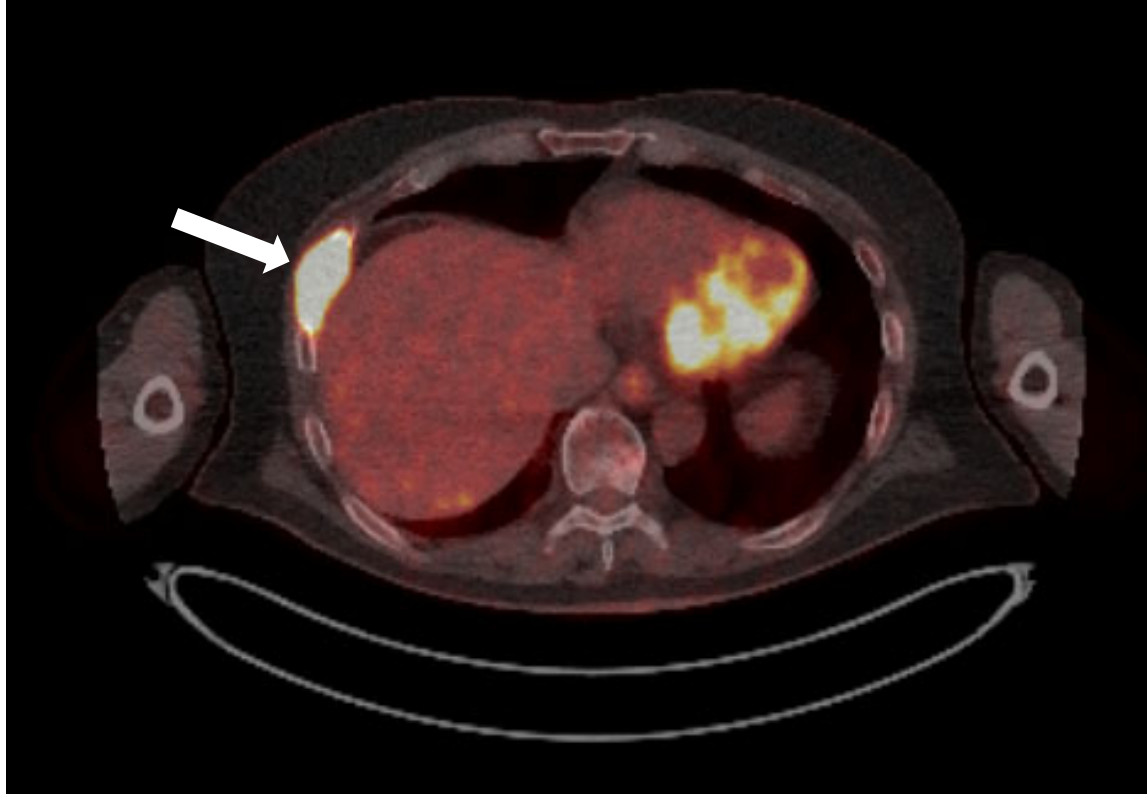

Supplement: Supplementary file 3 [file DataSheet_3.pdf]

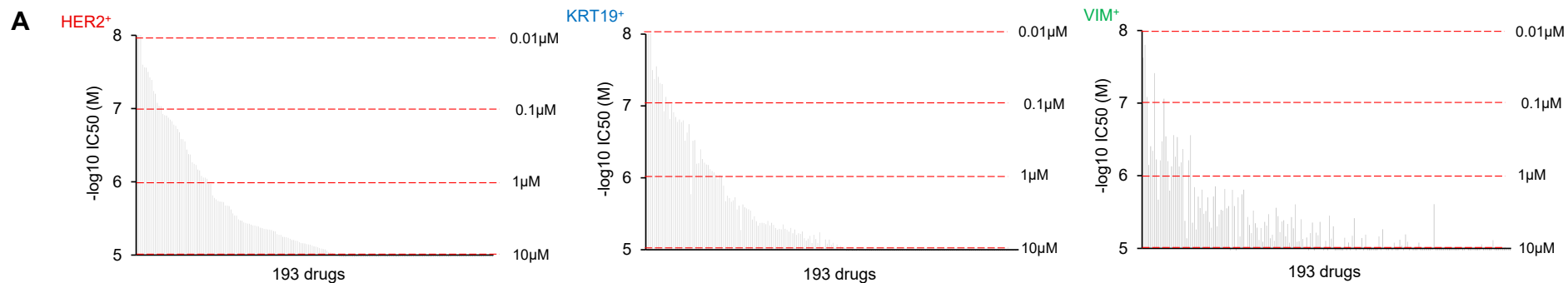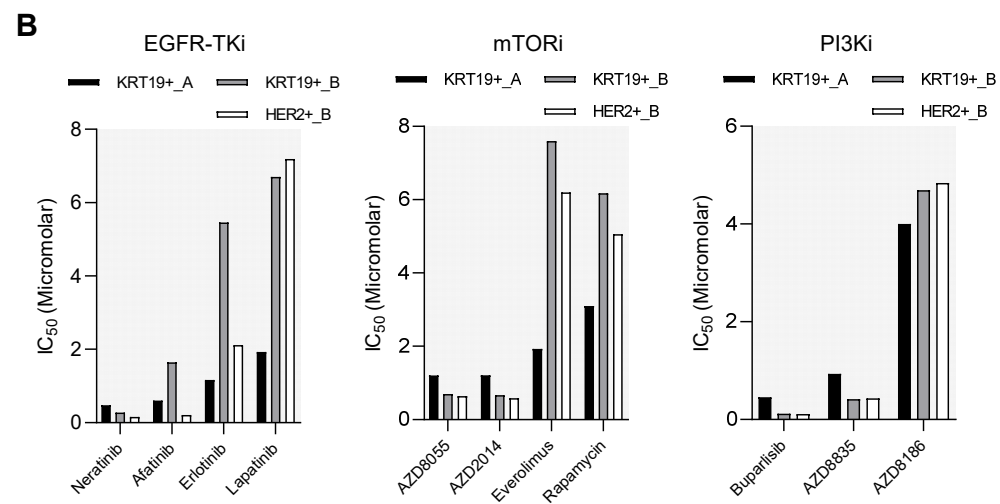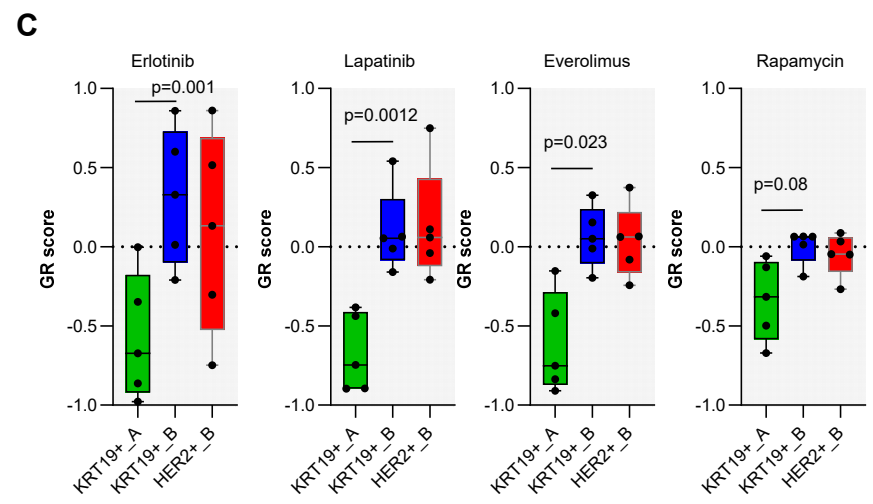

Supplement: Supplementary file 4 [file DataSheet_4.pdf]

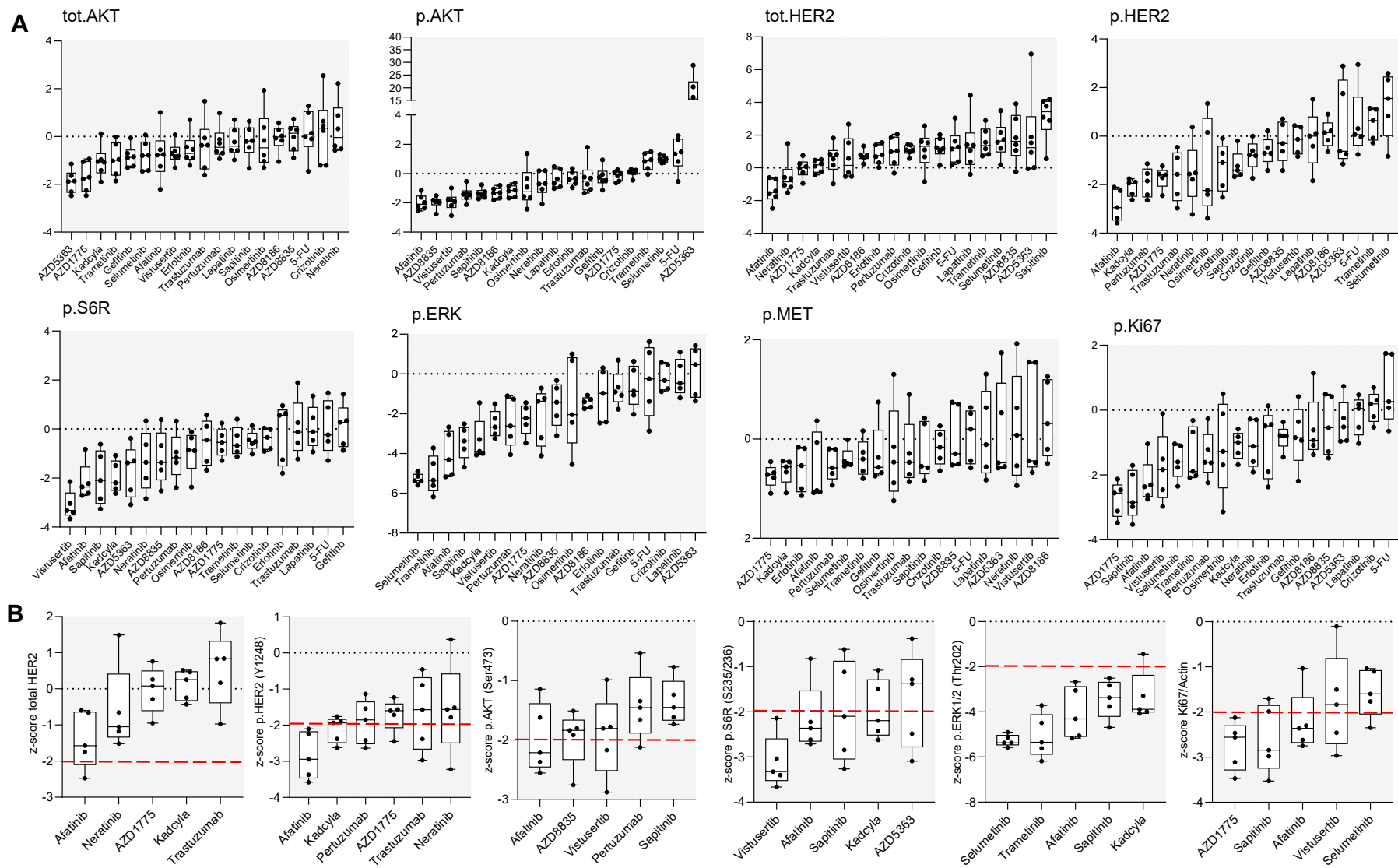

Supplement: Supplementary file 5 [file DataSheet_5.pdf]

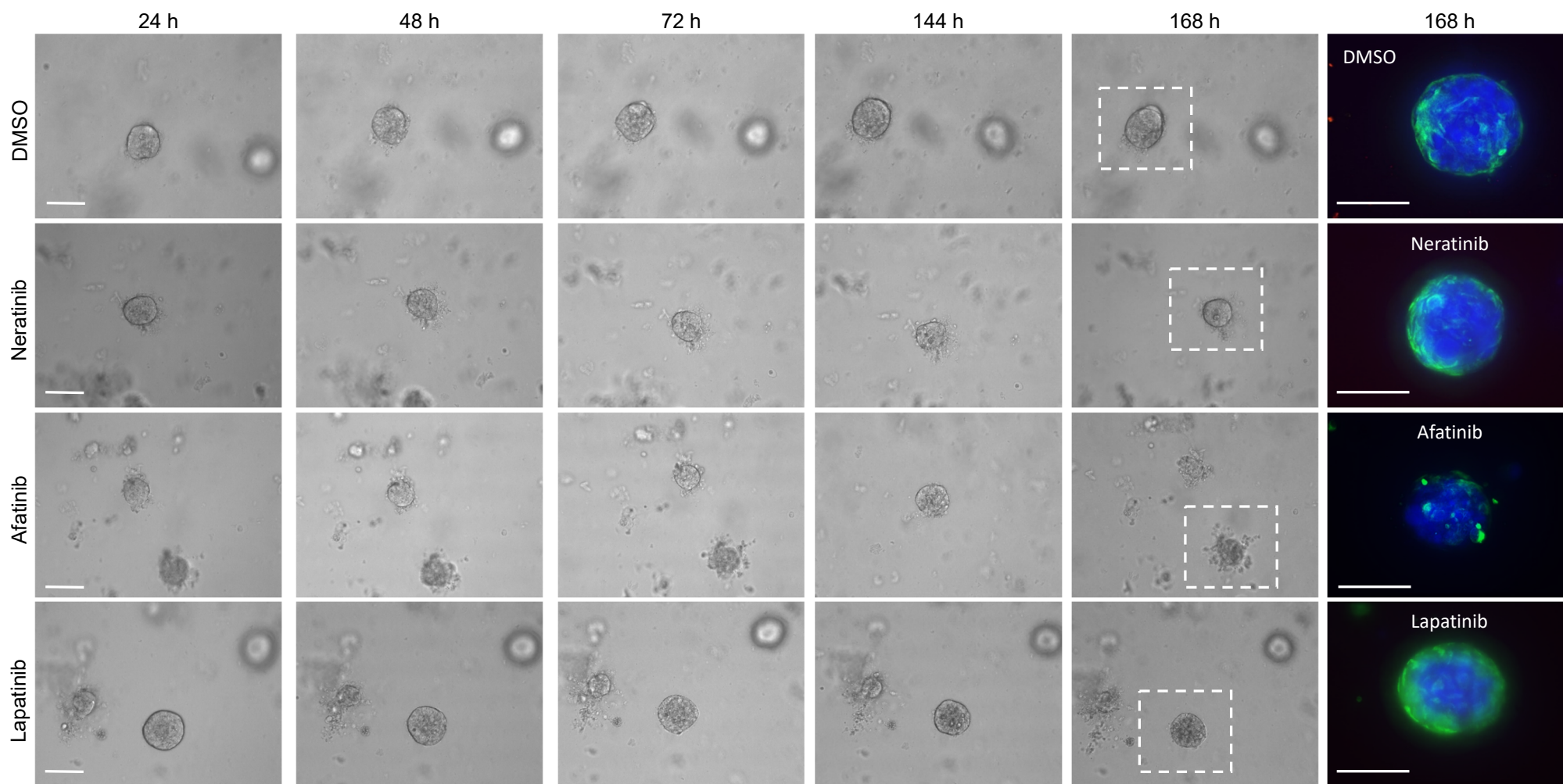

Supplement: Supplementary file 6 [file DataSheet_6.pdf]
